# Increased hemorrhagic transformation and altered infarct size and localization after experimental stroke in a rat model type 2 diabetes

**DOI:** 10.1186/1471-2377-7-33

**Published:** 2007-10-15

**Authors:** Adviye Ergul, Mostafa M Elgebaly, Mary-Louise Middlemore, Weiguo Li, Hazem Elewa, Jeffrey A Switzer, Christiana Hall, Anna Kozak, Susan C Fagan

**Affiliations:** 1 Program in Clinical and Experimental Therapeutics, College of Pharmacy, University of Georgia, USA; 2 Vascular Biology Center, Medical College of Georgia, USA; 3 Department of Neurology, Medical College of Georgia, USA; 4 Specialty Care Service Line, Veterans, Administration Medical Center, Augusta, GA, USA

## Abstract

**Background:**

Interruption of flow through of cerebral blood vessels results in acute ischemic stroke. Subsequent breakdown of the blood brain barrier increases cerebral injury by the development of vasogenic edema and secondary hemorrhage known as hemorrhagic transformation (HT). Diabetes is a risk factor for stroke as well as poor outcome of stroke. The current study tested the hypothesis that diabetes-induced changes in the cerebral vasculature increase the risk of HT and augment ischemic injury.

**Methods:**

Diabetic Goto-Kakizaki (GK) or control rats underwent 3 hours of middle cerebral artery occlusion and 21 h reperfusion followed by evaluation of infarct size, hemorrhage and neurological outcome.

**Results:**

Infarct size was significantly smaller in GK rats (10 ± 2 vs 30 ± 4%, p < 0.001). There was significantly more frequent hematoma formation in the ischemic hemisphere in GK rats as opposed to controls. Cerebrovascular tortuosity index was increased in the GK model (1.13 ± 0.01 vs 1.34 ± 0.06, P < 0.001) indicative of changes in vessel architecture.

**Conclusion:**

These findings provide evidence that there is cerebrovascular remodeling in diabetes. While diabetes-induced remodeling appears to prevent infarct expansion, these changes in blood vessels increase the risk for HT possibly exacerbating neurovascular damage due to cerebral ischemia/reperfusion in diabetes.

## Background

Ischemic stroke is a leading cause of death and disability in the United States and diabetes is the most rapidly increasing risk factor for stroke. Among patients with recent stroke, 70% have overt diabetes or prediabetes characterized by impaired fasting glucose or impaired glucose tolerance [1]. Type 2 diabetes, a disease that affects more than 17 million Americans with an alarming number of new cases, holds a 2–6 fold increased risk for stroke. Not only is the incidence of stroke increased among diabetics, but stroke patients with diabetes have a worse outcome. Mortality is increased in diabetics at one week, one month and three months after stroke, and diabetic stroke survivors have more profound neurologic deficits and disability [2]. In addition, diabetes and hyperglycemia predict early neurologic deterioration following ischemic stroke [3]. A recent study reported that persistent post-stroke hyperglycemia causes infarct expansion and worse clinical outcome [4]. Vascular complications of diabetes characterized by vascular dysfunction and pathological remodeling contribute to increased cardiovascular mortality and morbidity in diabetes, yet, changes in the cerebrovascular structure remain unknown.

During focal cerebral ischemia, damage to cerebral blood vessels occurs early and in a progressive fashion [5]. Reperfusion through damaged cerebral blood vessels is likely to further increase the ultimate tissue damage due to ischemic stroke. If ischemia is prolonged, cerebral edema occurs and bleeding into the brain parenchyma known as hemorrhagic transformation (HT) ensues and worsens stroke outcome. In experimental transient middle cerebral artery occlusion (MCAO) models, acute hyperglycemia induced by glucose administration augments ischemic injury and increases HT [6-8]. However, the impact of chronic mild hyperglycemia as seen in majority of Type 2 diabetic patients on cerebral vessel structure and mechanisms and functional consequences especially in the acute ischemia/reperfusion injury setting are unclear. The current study tested the hypothesis that diabetes-induced changes in the cerebral vasculature increase the risk of HT and augment ischemic injury.

## Methods

### Animal and tissue preparation

GK rats, a spontaneous model of Type2 diabetes generated from selective inbreeding of glucose-intolerant Wistar rats, developed hyperglycemia at 6-weeks of age and had been diabetic for almost 4–5 weeks prior to stroke surgery. Regular Wistar rats served as control as reported [9]. All experiments were performed on male Wistar (Harlan, Indianapolis, IN) and Goto-Kakizaki (in-house bred, derived from the Tampa colony) rats (n = 5–6/group) within a narrow range of body weight (250–290 g) [10]. All protocols were approved by the Institutional Animal Care and Use Committee at the VA Medical Center. Animals were allowed access to food and water ad libitum, and were maintained on a 12/12 hour light/dark cycle. During housing, drinking water measurements, weight, and blood glucose measurements were performed twice weekly. Glucose measurements were taken from the tail vein and measured on a commercially available glucose meter (*AccuChek*, Roche Diagnostics, Indianapolis, IN) [9].

### Experimental cerebral ischemia

All animals were anesthetized with 2% isoflurane via inhalation. Focal cerebral ischemia was induced using the intraluminal suture MCAO model as previously reported by us and others [11-13]. The right MCA was occluded with a 19–21 mm 3-0 surgical nylon filament, which was introduced from the external carotid artery lumen into the internal carotid artery to block the origin of the MCA. A significant drop in blood flow as measured by laser Doppler (Perimed, North Royalton, OH) indicated successful occlusion of the MCA. A similar degree of reduction in blood flow was achieved in both control and GK rats. The suture was removed after 3 hours of occlusion and measurement of blood flow was repeated to determine whether the flow was restored after reperfusion which appeared to be similar in both groups. The animals were then returned to their cages.

### Neurologic assessment

Neurologic function was quantified prior to reperfusion and at 24 hours (just prior to sacrifice) using the Bederson score [11,12]. An animal with no apparent deficits obtains a 0 on the assessment and a score of 3 is consistent with a middle cerebral artery occlusion. Only animals with a score of 3 prior to reperfusion were included in the analysis.

### Assessment of infarct size and hemorrhage

At 24 hours after the onset of MCAO, blood glucose was measured and then the animals were anesthetized with ketamine 44 mg/kg and xylazine 13 mg/kg I.M. (cocktail), perfused with saline, sacrificed and brains were removed. The brain tissue was sliced into seven 2-mm thick slices in the coronal plane. HT was defined as the presence of visible bleeding in coronal brain sections prior to staining. Slices were then stained with a 2% solution of 2, 3, 5-triphenyltetrazolium chloride (TTC) (Sigma Chemical Co., USA) for 15–20 minutes. Images of the stained sections were taken. Grossly visible infarction zones were quantified using image analysis software (Zeiss-KS300) [11-13]. The infarct volume was determined as % the contralateral hemisphere to correct for edema.

### Visualization of cerebrovasculature and measurement of tortuosity index

Additional control and GK animals (250–290 g, n = 5/group) were anesthetized with sodium pentobarbital and injected with 40 mg/kg papaverine hydrochloride to induce maximal vasodilatation as previously described [14]. The thoracic aorta was clipped and 2 ml of warm (37°C) latex (Vultex, Chicago Latex Products No. 563) mixed with carbon black was injected through the cannula over a 2-minute period. 15 minutes after the injection, rats were decapitated, brains were removed and placed in cold saline. Images of the top and bottom of the brains were digitized, divided into 6 regions with a grid and in each section 2 middle size vessels were traced on a Wacom tablet 493-3 using Image J I-36 software. Tortuosity index (TI) was defined as the ratio of the vessel length over straight line distance between two vessel ends. Then the average of 24 measurements was used as the TI per animal.

### Vessel morphometry

MCAs were perfused with Histogel (Richard Allen Scientific, Kalamazoo, MI), then excised and embedded in the same matrix. Upon gelling of the matrix, the embedded vessel was placed in 10% formalin, embedded in paraffin, sectioned at 4 microns and mounted on treated slides. Sections were stained with Masson trichrome stain. Slides were viewed using a Zeiss Axiovert microscope (Carl Zeiss, Inc., Thornwood, NY) and media:lumen ratios (M/L) were analyzed using Spot software (Diagnostic Instruments, MI). 4 measurements were made per section and each animal had at least 3 sections as we reported previously [9].

### MMP activity

MCAs were homogenized and processed for MMP activity by gelatin zymography as we previously reported [9]. Recombinant MMP-2 protein (Calbiochem, San Diego, CA) was run in parallel with all samples and the band intensity on zymogram gels was normalized to that of standard to prevent gel-to-gel variability. Gelatinolytic activity was assessed by densitometric analysis (Gel-Pro version 3.1, Media Cybernetics, Carlsbad, CA)

### Statistical analysis

Comparisons of tortuosity index and infarct size between the two groups of rats were made using nonparametric Student's t-test. HT was analyzed by Fisher's exact test. Results are expressed as mean ± SEM. Effects were considered statistically significant at p < 0.05.

## Results

**Figure 1:**
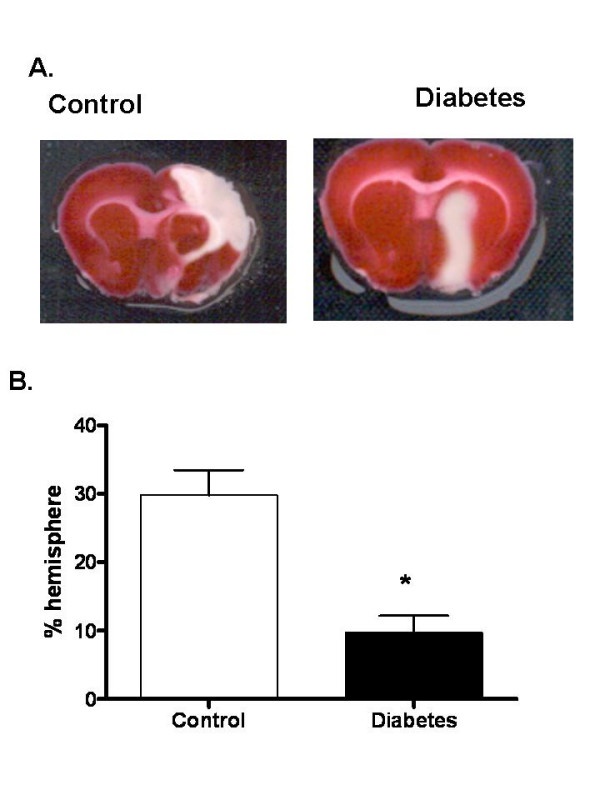
Infarct size is reduced in diabetes. (A) A representative image showing TTC staining for infarct size and localization in control Wistar (n = 10) and diabetic GK rats (n = 9). (B) Bar graph depicting infarct size in all the animals. Results are given mean ± sem and *p < 0.05 vs control.

Average blood glucose of GK rats prior to surgery was 170 ± 6 versus 110 ± 6 mg/dl in control animals and GK rats had been diabetic for 4–5 weeks prior to surgery. Mean arterial blood pressure prior to MCAO was 112 ± 5 and 110 ± 3 mmHg in control and GK groups. The metabolic profile of the colony used in this study was published previously [9,15]. As shown in a representative image in Fig. 1 Panel A, in all GK rats a polyp shape infarct was localized to subcortical area as opposed to the infarct occupying almost the entire hemisphere in control rats. In addition, infarct size was significantly smaller in the diabetic group. Edema as assessed by % change from the non-ischemic hemisphere was significantly less in GK rats (12.7 ± 1.5 vs 5.3 ± 0.6, p = 0.007).

**Figure 2:**
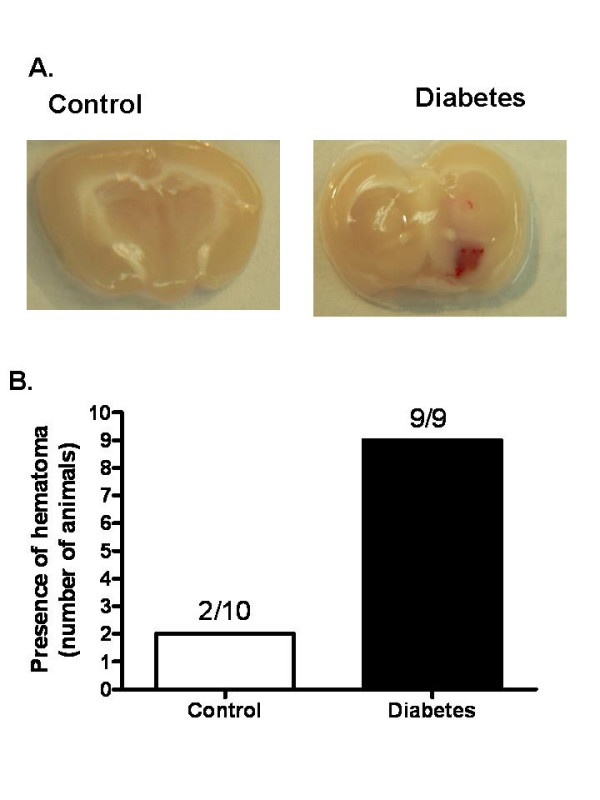
Incidence of HT is increased in diabetes. (A) A representative image showing visible hemorrhage in control Wistar (n = 10) and diabetic GK rats (n = 9). (B) Bar graph depicting incidence of HT in all the animals. Results are given mean ± sem and *p < 0.05 vs control.

In all GK rats (n = 9) there was overt hemorrhage in the subcortical sections where HT is usually observed (Fig. 2). In the control group, only 2 animal (n = 10) presented with bleeding. There was no difference in neurological outcome as determined by Bederson scale (2.6 ± 0.2 vs 2.9 ± 0.1).

**Figure 3:**
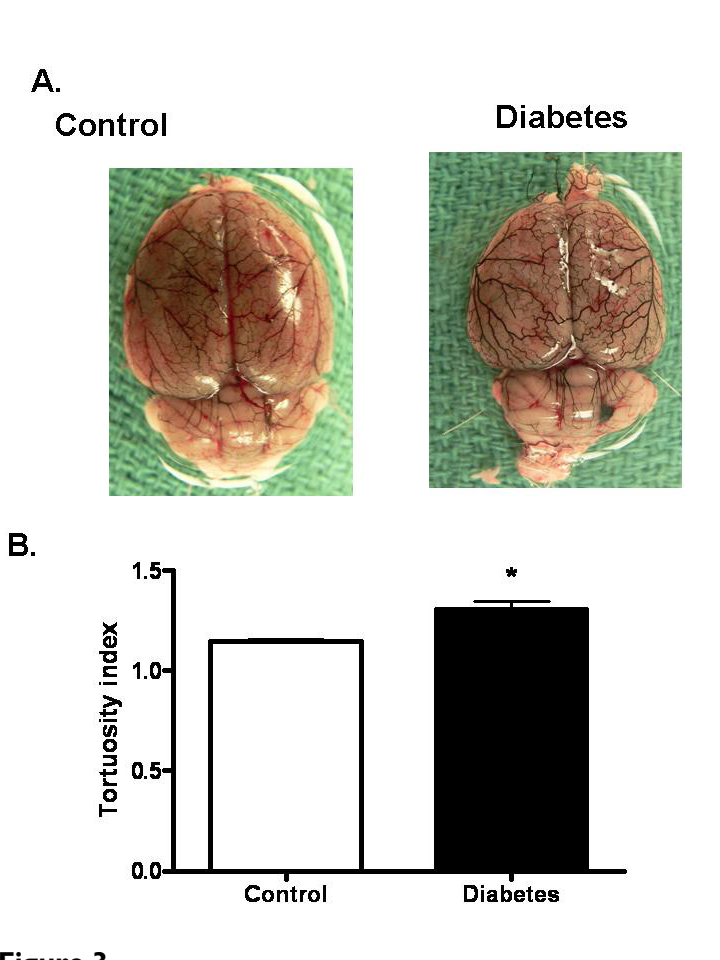
Increased tortuosity as index of vascular remodeling in diabetes. (A) A representative image showing superficial cerebral vessels in control Wistar (n = 5) and diabetic GK rats (n = 4), and (B) Bar graph summarizing results of TI measurements in all the animals. Results are given mean ± sem and *p < 0.05 vs control.

**Figure 4:**
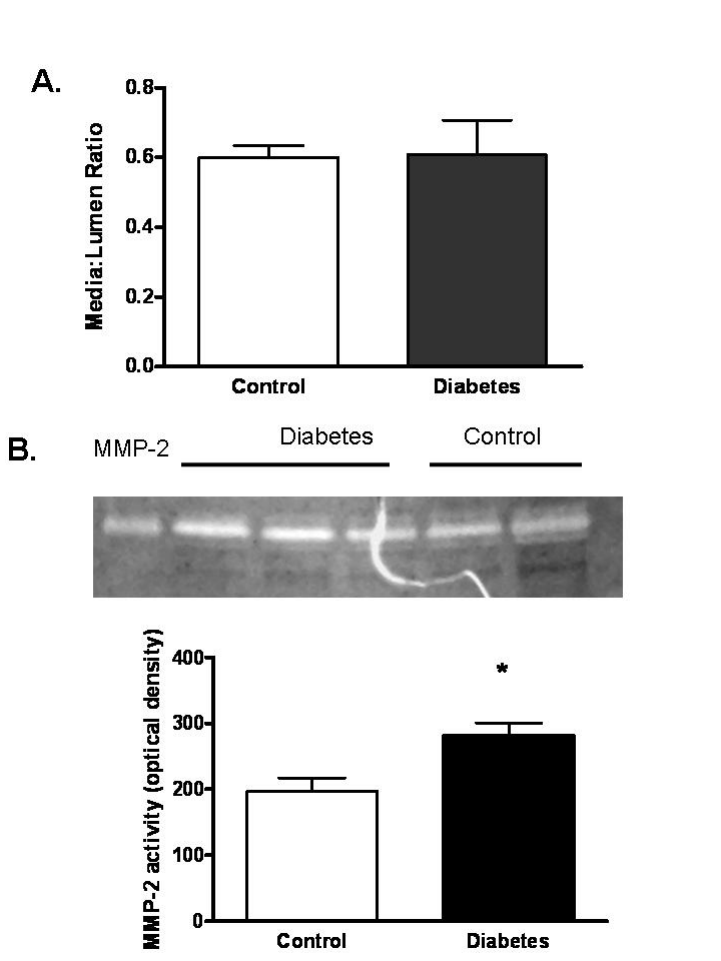
Increased MMP activity in diabetes. (A) Vessel segments were analyzed for morphological changes and collagen deposition by Masson staining which did not show any difference between control and diabetes groups. (B) A representative zymogram showing changes in vascular MMP-2 activity and densitometric analysis of lytic bands from all samples indicates an increase in MMP-2 activity. Results are given mean ± sem (n = 5–9) and *p < 0.05 vs control.

In order to determine whether these differences in infarct localization arise from anatomical differences of vessels that are occluded during stroke surgery and also evaluate the vascular structure, the cerebrovascular tree was visualized with latex/carbon black mixture. In GK rats, TI was significantly increased (1.13 ± 0.01 vs 1.34 ± 0.06, 19%) indicative of increased vascular density and remodeling (Fig. 3A and 3B). Images of Circle of Willis and branching arteries including MCA revealed no macroscopic differences between control and GK rats. Morphometric analysis of MCAs isolated from additional animals (n = 5–9) that were not exposed to MCAO displayed similar medial thickness and lumen diameter (Fig. 4A). On the other hand, MMP-2 activity of isolated MCAs was significantly increased in the diabetic group (Fig. 4B).

## Discussion

This study questioned whether and to what extent diabetes-induced changes in the cerebral vasculature increase the risk of hemorrhage and augment ischemic injury. There were 3 important findings in this study. First, infarct size was smaller and localization was different in the diabetic rats. Second, hemorrhagic transformation was increased in this group. Third, despite a very short duration of mild diabetes, tortuosity of cerebral vessels was significantly enhanced. These results provide important and timely information regarding the potential mechanisms that may contribute to increased stroke risk and worse outcome in diabetes.

In recent years, the role of hyperglycemia in the pathophysiology and outcome of acute ischemic stroke has gained significant attention. Numerous studies including the NINDS tPA stroke trial showed that elevated admission blood sugar was a significant predictor of poor clinical outcome and HT [16-18]. The relative role of acute versus chronic hyperglycemia in the pathogenesis of this poor outcome has been debated and has not been determined unequivocally [10]. However, based on the findings that short-term mortality is higher and final infarct size is larger in nondiabetic patients who present with hyperglycemia at admission, several studies have suggested that hyperglycemia and not necessarily diabetes aggravates ischemic injury and stroke outcome [18,19]. In experimental models, hyperglycemia has been shown to increase infarct size and a limited number of studies also demonstrated augmented HT in hyperglycemic ischemia/reperfusion injury [6-8]. However, these studies employed hyperglycemia induced by glucose injection prior to or at the time of MCA and blood glucose levels were above 300 mg/dl. The intriguing finding of the current study is that infarct size is smaller but secondary hemorrhage is larger in diabetic GK rats that present with moderate elevations in blood glucose (180–250 mg/dl). Consistent with smaller infarct size, diabetic rats had less edema. Moreover, the infarct is subcortical as opposed to subcortical and cortical localization in control rats. These results suggest a difference in the pathophysiology of ischemic injury in the diabetic state. It is well established that diabetes promotes ischemic preconditioning in the myocardium [20]. It is also known that ischemic preconditioning is neuroprotective [21,22]. It is possible that in our model, diabetes induced-changes in the cerebrovasculature result in ischemic preconditioning and thereby prevent infarct expansion but promote BBB breakdown and hemorrhage emphasizing the importance of the neurovascular unit.

During focal cerebral ischemia, disruption of the BBB complex, which consists of the endothelial cell, its tight junctions, the basal lamina and the astrocytic foot processes results in damage to the entire neurovascular unit [23-25]. If the ischemia is prolonged, breakdown of BBB increases cerebral injury by the development of vasogenic edema and secondary hemorrhage known as HT [11,26]. Matrix metalloproteinases (MMPs) are a class of zinc-dependent endopeptidases that contribute to the breakdown of BBB. Several laboratories have demonstrated that MMP activity, especially gelatinases MMP-2 and MMP-9, is increased after focal cerebral ischemia and contributes to the development of HT [23,25,27,28]. MMPs are also up-regulated in diabetes. We have also found that MMP activity is increased in MCAs of GK rats and this increase is associated with enhanced pathological remodeling after 10–12 weeks of diabetes [9]. The animals used in the current study had mild diabetes only for 4–5 weeks. While we cannot rule out the possibility that in-breeding may cause alterations in vessel structure, reactivity and mechanics that may contribute to the changes we observe in this colony of diabetic rats, our data provide evidence that despite the short duration of diabetes, there was increased MMP-2 activity and tortuosity suggesting early cerebrovascular remodeling. This remodeling response may contribute to increased hemorrhage in diabetes.

Increased tortuosity is commonly measured in evaluation of coronary vessels. This is an indicator of ischemia-induced vascular restructuring as it increases vessel surface area. The current study found that the TI of cerebral vessels was increased in diabetes. These findings suggest that there is microvascular remodeling and potential neovascularization in the GK rat, which may contribute to decreased infarct size. Neovascularization in diabetes is very complex and regulated in a temporal and tissue specific manner. It is well established that hyperglycemia-mediated oxidative damage to microvascular endothelial cells triggers a cascade of events that lead to changes in vascular proliferative retinopathy and formation of new blood vessels on the surface of the retina [29,30]. These immature vessels then break and leak worsening the retinal damage. Although we do not have direct evidence, it is highly possible that similar to retina, in the cerebrovasculature newly formed or remodeled vessels cannot resist the impact of ischemia/reperfusion injury and increased bleeding occurs. Preliminary evaluation of baseline BBB permeability in the absence of ischemic injury by Evans blue extravasation method was not sensitive to detect permeability differences between control and diabetic animals. Interestingly, edema after MCAO was significantly less in GK rats most likely due to smaller infarct size. It has to be recognized that the current study has several limitations. First, cerebral blood flow was measured to ensure that similar degree of blood flow is achieved in control versus diabetic rats during MCAO and after reperfusion but evaluation of blood flow with more reliable methods are needed to assess whether changes observed in vessel structure are associated with alterations in baseline blood flow as well as collateral flow during MCAO. Second, HT was defined as the presence of visible bleeding in coronal brain sections and more quantitative methods are needed. Similarly, vascular remodeling was assessed by visualization of the vascular tree, measurement of tortuosity and morphometry of histogel and not pressure-fixed vessels. Whether increased tortuosity is due to remodeling of existing vessels or due to neovascularization or both remains to be determined. Lastly, the effect(s) glycemic control on the severity of ischemic injury and outcome remain to be determined. Nevertheless our findings are very important and timely.

## Conclusion

Given that thrombolytic therapy to open the occluded artery is the best chance to recover from ischemic damage and that admission blood glucose levels predict whether HT will complicate the thrombolytic therapy, better understanding of the mechanisms underlying increased bleeding in diabetes will identify novel therapeutic targets and strategies. The results of this study suggest that different mechanisms may contribute to ischemic damage in hyperglycemia versus diabetes.

## Competing interests

The author(s) declare that they have no competing interests.

## Authors' contributions

AE was involved in all aspects of the study, MME, HE and WL assisted with surgeries and analyses of infarct data, M-LM conducted tortuosity studies, JAS and CH contributed to the preparation of the manuscript, AK performed MCAO experiments, and SCF contributed to data analyses and manuscript preparation. All authors approved the final version of the manuscript.

## Pre-publication history

The pre-publication history for this paper can be accessed here:


